# Common Genetic Variation near MC4R Has a Sex-Specific Impact on Human Brain Structure and Eating Behavior

**DOI:** 10.1371/journal.pone.0074362

**Published:** 2013-09-16

**Authors:** Annette Horstmann, Peter Kovacs, Stefan Kabisch, Yvonne Boettcher, Haiko Schloegl, Anke Tönjes, Michael Stumvoll, Burkhard Pleger, Arno Villringer

**Affiliations:** 1 Max-Planck-Institute for Human Cognitive and Brain Sciences, Leipzig, Germany; 2 IFB Adiposity Diseases, University of Leipzig, Germany; 3 Interdisciplinary Center of Clinical Research, University of Leipzig, Leipzig, Germany; 4 Department of Medicine, University of Leipzig, Germany; 5 Day Clinic of Cognitive Neurology, University of Leipzig, Germany; 6 Mind and Brain Institute, Berlin School of Mind and Brain, Humboldt-University, Berlin, Germany; University of Jaén, Spain

## Abstract

Obesity is associated with genetic and environmental factors but the underlying mechanisms remain poorly understood. Recent genome-wide association studies (GWAS) identified obesity- and type 2 diabetes-associated genetic variants located within or near genes that modulate brain activity and development. Among the top hits is rs17782313 near MC4R, encoding for the melanocortin-4-receptor, which is expressed in brain regions that regulate eating. Here, we hypothesized rs17782313-associated changes in human brain regions that regulate eating behavior. Therefore, we examined effects of common variants at rs17782313 near MC4R on brain structure and eating behavior. Only in female homozygous carriers of the risk allele we found significant increases of gray matter volume (GMV) in the right amygdala, a region known to influence eating behavior, and the right hippocampus, a structure crucial for memory formation and learning. Further, we found bilateral increases in medial orbitofrontal cortex, a multimodal brain structure encoding the subjective value of reinforcers, and bilateral prefrontal cortex, a higher order regulation area. There was no association between rs17782313 and brain structure in men. Moreover, among female subjects only, we observed a significant increase of ‘disinhibition’, and, more specifically, on ‘emotional eating’ scores of the Three Factor Eating Questionnaire in carriers of the variant rs17782313’s risk allele. These findings suggest that rs17782313’s effect on eating behavior is mediated by central mechanisms and that these effects are sex-specific.

## Introduction

Obesity is associated with genetic and environmental factors but the underlying mechanisms are still to be elucidated. Recent genome-wide association studies (GWAS) identified several obesity- and type 2 diabetes-associated genetic variants located within or near brain-expressed genes that moderate brain activity and its development [1]. One of the two strongest association signals lies near *MC4R*, encoding for the melanocortin-4-receptor, which is predominantly expressed in brain regions that regulate eating. MC4R, together with MC3R, α-melanocyte-stimulating hormone (α-MSH) and Agouti Related Peptide (AGRP) comprise the central part of the melanocortin system which modulates complex physiological processes such as energy homeostasis, sexual function, learning, anxiety, anticipatory behavior, stress reactivity, and circadian rhythm [2]. Furthermore, they have been implicated in both the acute regulation of satiety and feeding behavior and in the integration of long-term appetitive signals [3].

Mutations within *MC4R* lead to massive hyperphagia and severe, monogenic early onset obesity in humans [4]. In rodent brains, MC4R expression has been confirmed in amygdala, hippocampus, hypothalamus, orbital cortex, olfactory cortex, striatum and brainstem [5], with dimorphic sexual differences in some of these regions. However, the distribution of MC4 receptors in the human brain is not known to date.

rs17782313, a single nucleotide polymorphism (SNP), mapping to a locus 188kb downstream of *MC4R*, is speculated to participate in the function and/or expression of MC4R and the minor allele (C, minor allele frequency about 27% in white Europeans) is associated with a higher prevalence of common human obesity in genome-wide association studies (GWAS) [1], [6]–[9], with increased occurrence of snacking behavior [10], and a higher hunger score on the Three-Factor Eating Questionnaire (TFEQ [11]). An association with higher energy and fat intake was reported [12] but could not be confirmed [13]. Each copy of the rs17782313 minor (C) allele is associated with an increase in BMI of ∼0.22 kg/m^2^ [7] in adults. Sex has repeatedly been shown to affect the association between rs17782313 and obesity markers, showing a more pronounced effect for women in three out of four studies [8], [14]–[16].

Here, we examined effects of this common genetic variant near *MC4R* both on eating behavior and brain structure. We hypothesized that (1) rs17782313 is associated with changes in human eating behavior and (2) that these are associated with structural alterations in eating-related brain regions, specifically those known to express MC4R. Further, we hypothesize possible gender differences in rs17782313’s impact on eating behavior and brain structure.

## Methods

### Subjects

221 healthy volunteers (105 female, age 26 years, SD 5, BMI 27.1 kg/m^2^, SD 7.1) were examined (for demographic details see Table 1 [whole sample] & Table 2[split by gender]). Participants were recruited in Leipzig, Germany, via announcements or the local participant database of the Max Planck Institute for Human and Cognitive Brain Sciences. All participants were white, right-handed dominant and non-smoking. None reported a history of medical, neurological or psychiatric disease, head injuries, or smoking, and none were on medications that may affect brain function, appetite or body weight. Normal levels of thyroid hormones were observed for each subject. Participants gave written informed consent prior to the measurements and were paid €8 per hour for their participation. The local ethics committee of the University of Leipzig approved the study.

**Table 1:** Detailed demographic and questionnaire data split by rs17782313 near MC4R.

		rs17782313			
**Neuroimaging**					
MAF		CC (n = 15)	AC (n = 87)	AA (n = 119)	p^a^
0.265	Age [years]	27 (5) *[24–28.99]*	25 (5) [*24]*–[*26*]	25 (5) [*24]*–[*26*]	.55
	BMI [kg/m^2^]	25.7 (9.9) *[23.1–32.5]*	25.1 (9.2) *[24.1–28.3]*	23.9 (9.5) *[23.2–25.5]*	.46
**TFEQ-51 & Neuroimaging**					
0.277	n	14	74	97	p^a^
	Age [years]	27 (5) [*24]*–[29]	25 (5) [*24]*–[26]	25 (6) [*24]*–[26]	.448
	BMI [kg/m^2^]	28.05 (9.9) *[23.4–32.6]*	25.65 (9.2) *[24–29.4]*	24.40 (10.5) *[23.3–25.9]*	.444
					p^b^
	TFEQ-51 Cognitive Restraint*	8 (5) [5*]*–[10]	5.5 (7) [4*–*6.5]	5 (6) [4*]*–[6]	.237
	TFEQ-51 Disinhibition*	**9 (6)** [6*]*–[11]	**5 (3)** [5*]*–[6]	**5 (4)** [5*]*–[6]	**F_(2,177) = _8.34, p<.001** [one-way ANOVA]
	TFEQ-51 Hunger	6.5 (6) [3*]*–[9]	5 (4) [4]–[6]	5 (5) [4.5*–*6]	.510
**TFEQ-R18 & Neuroimaging**					
0.256	n	14	54	92	p^a^
	Age [years]	27 (5) [*24]*–[29]	26 (5) [*24]*–[27]	25 (6) [*24]*–[26]	.457
	BMI [kg/m^2^]	28.05 (9.9) *[23.4–32.6]*	25.45 (8.6) *[24–28.9]*	24.40 (10.9) *[23.3–27.2]*	.476
					p^b^
	TFEQ-18 Cognitive Restraint^#^	2 (2) [1*]*–[3]	1 (3) [0*–*2]	1 (2) [1*–*1]	.214
	TFEQ-18 Uncontrolled Eating	3.5 (4) [2*]*–[6]	3 (3) [1.5*–*3]	3 (4) [2*–*3.5]	.279
	TFEQ-18 Emotional Eating*	1 (3) [0*–*3]	0 (2) [0*–*1]	0 (1) [0*–*0]	.140

Data are presented as medians and interquartile range in round brackets, 95% confidence intervals of the median based on 1,000 bootstrap samples are given in squared brackets. n: number of participants per category, TFEQ 51: Three Factor Eating Questionnaire, TFEQ R18: Revised and shortened version of the TFEQ, MAF: Minor Allele Frequency (describes the fraction of the minor allele, C, considering the sum of all alleles), p^a^: p-value of Kruskal-Wallis one-way analysis of variance, p^b^: p-value of Kruskal-Wallis one-way analysis of variance on residuals after correcting for age and BMI. * =  significant (p<.05) association with BMI in linear regression model, ^#^ = significant (p<.05) association with age in linear regression model.

**Table 2:** Detailed demographic and questionnaire data split by gender and rs17782313 near MC4R.

		female (*n = 105*)					male (*n = 116*)			
**Neuroimaging**										
MAF		CC	AC	AA	p^a^	MAF	CC	AC	AA	p^a^
*0.26*	*n*	*7*	*40*	*58*		*0.27*	*8*	*47*	*61*	
	Age [years]	26 (7) *[22*–*34.5]*	24 (4) *[23.5*–*26]*	24.5 (6) [*23*]–[*26*]	0.71		27 (5) *[24*–*30]*	25 (6) [*24*]–[*27*]	25 (6) [*24*]–[*27*]	0.81
	BMI [kg/m^2^]	25.7 (10.7) *[21.9*–*32.6]*	24.5 (9.4) *[22.3*–*28.3]*	23.9 (10.1) *[22.3*–*27.7]*	0.74		28.51 (10.6) *[23.4*–*34.5]*	25.5 (9.4) *[24.2*–*30.3]*	24.21 (10) *[23.1*–*25.5]*	0.54
**TFEQ-51 & Neuroimaging**										
*0.28*	*n*	*6*	*29*	*38*	p^a^	*0.27*	*8*	*45*	*59*	p^a^
	Age [years]	27 (8) *[23.5*–*35]*	26 (5) [*23*]–[*26*]	25 (7) *[23*–*26.5]*	0.52		27 (5) *[24*–*30]*	25 (6) [*24*]–[*27*]	25 (5) [*24*]–[*27*]	0.82
	BMI [kg/m^2^]	28.05 (11) *[22.7*–*34.3]*	25.5 (9.9) *[21.6*–*29.8]*	24.3 (10.7) *[22.2–28.4]*	0.60		28.51 (10.6) *[23.4*–*34.5]*	25.8 (9.5) *[24.2*–*30.6]*	24.5 (10.2) *[23.2*–*25.5]*	0.57
					*p^b^*					*p^b^*
	TFEQ-51 Cognitive Restraint	8.5 (7) [*4*]–[*12*]	8 (9) [*6*]–[*11*]	6 (10) *[3*–*9.5]*	>.15		7 (5) [*5*]–[*11*]	4 (5) [*3*]–[*5*]	5 (5) [*3*]–[*6*]	0.105
	TFEQ-51 Disinhibition*	**11.5 (4)** *[8.5*–*13]*	**6 (5)** [*5*]–[*8*]	**6 (5)** [*4]*–[7]	**0.001**	*	7.5 (6) [*3*]–[*9*]	5 (3) [*4*]–[*6*]	5 (4) [*4*]–[*6*]	>.15
	TFEQ-51 Hunger	7 (5) *[3*–*8.5]*	4 (5) [*3*]–[*6*]	5 (5) [*4*]–[*7*]	>.15		5 (8) [*3*]–[*11*]	5 (4) [*5*]–[*6*]	6 (5) [*4*]–[*7*]	>.15
**TFEQ-R18 & Neuroimaging**										
*0.26*	*n*	*6*	*24*	*38*	p^a^	*0.25*	*8*	*31*	*54*	p^a^
	Age [years]	27 (8) *[23.5*–*35]*	26 (4) [*23*]–[*26*]	25 (7) *[23*–*26.5]*	0.51		27 (5) *[24*–*30]*	26 (5) [*24*]–[*27*]	25.5 (5) [*24*]–[*27*]	0.81
	BMI [kg/m^2^]	28.05 (11) *[22.3*–*34.3]*	26.85 (9.1) *[22.3*–*30.1]*	24 (10.6) *[22.2*–*28.4]*	0.66		28.51 (10.6) *[23.4*–*34.5]*	24.9 (8.7) *[23.5*–*28.9]*	24.75 (10.9) *[23.1*–*29.2]*	0.66
					*p^b^*					*p^b^*
	TFEQ-R18 Cognitive Restraint	2 (2) *[.5*–*3]*	2 (3) [*1*]–[*4*]	1 (3) *[.5*–*2]*	>.15	^#^	2 (2) [*1*]–[*4*]	0 (1) *[0*–*1]*	1 (2) *[0*–*1]*	0.022
	TFEQ-R18 Uncontrolled Eating	4 (4) *[2.5*–*6.5]*	2.5 (3) [*1*]–[*4*]	3 (4) [*2*]–[*4*]	>.15		3 (5) [*2*]–[*8*]	3 (3) [*1*]–[*4*]	3 (4) [*2*]–[*4*]	>.15
	TFEQ-R18 Emotional Eating*	**3 (0)** [*2*]–[*3*]	**1 (3)** *[0*–*2]*	**0 (2)** *[0*–*1]*	**0.001**	*	0 (1) *[0–1]*	0 (0) *[0*–*0]*	0 (0) *[0*–*0]*	>.15

Data are presented as medians and interquartile range in round brackets, 95% confidence intervals of the median based on 1,000 bootstrap samples are given in squared brackets. n: number of participants per category, TFEQ 51: Three Factor Eating Questionnaire, TFEQ R18: Revised and shortened version of the TFEQ, MAF: Minor Allele Frequency (describes the fraction of the minor allele, C, considering the sum of all alleles), p^a^: p-value of Kruskal-Wallis one-way analysis of variance, p^b^: p-value of Kruskal-Wallis one-way analysis of variance on residuals after correcting for age and BMI. * =  significant (p<.05) association with BMI in linear regression model, ^#^ = significant (p<.05) association with age in linear regression model.

### Genotyping

rs17782313 on chromosome 18 was genotyped by using the TaqMan allelic discrimination assay (Applied Biosystems, Inc.) on an ABI PRISM 7500 sequence detector (Applied Biosystems Inc.) according to the manufacturer’s protocol. The genotype distribution was consistent with Hardy-Weinberg equilibrium (*P-*value = 0.97). Genotyping success rate was 100%. To assess genotyping reproducibility, a random ∼5% selection of the sample was re-genotyped; all genotypes matched initial designated genotypes. With rs17782313 we chose the variant near *MC4R* with the strongest known association to obesity. The linkage disequilibria for this variant with other SNPs near *MC4R* are as follows: r^2^ = 1.0 between rs17782313 and rs571312, and r^2^ = 0.85 between rs571312/rs17782313 and rs12970134 [15].

### Three Factor Eating Questionnaire (TFEQ)

184 participants (72 women) completed the German version of the Three Factor Eating Questionnaire with 51 items (TFEQ-51 [11]). This questionnaire has three subscales, measuring three dimensions of human eating behavior. Higher values indicate a more pronounced expression of the respective behavior. ‘Cognitive Restraint of Eating’ (21 items) measures whether eating behavior is under cognitive, rather than physiological control, ‘Disinhibition of Eating’ (16 items) measures the lack of control over eating, especially in the presence of tempting external cues or situations, and ‘Susceptibility to Hunger’ (14 items) measures the experience of prominent and disturbing subjective hunger feelings. The number of items is identical with the highest possible score on each scale.

To further specify the aspects of eating behavior affected by rs17782313, we adopted the revised and recently validated scoring scheme of the TFEQ-R18 in addition to the original scoring theme, which relies on only 18 items of the original questionnaire and providing the subscales ‘Cognitive Restraint of Eating’ (six items, e.g. “I deliberately take small helpings as means of controlling my weight.”), ‘Uncontrolled Eating’ (nine items, e.g. “When I smell a delicious food, I find it very difficult to keep from eating, even if I have just finished a meal.”) and ‘Emotional Eating’ (three items, e.g. “When I feel blue, I often overeat.”) [17].

The TFEQ-51 and the TFEQ-R18 correspond to each other as follows. The TFEQ-51 has 51 items and three subscales: Cognitive Restraint, Disinhibition and Hunger. The TFEQ-R18 has been derived from the TFEQ-51 using a subset of the original items which however were allocated (according to a factor analysis) into partly different subscales: Cognitive Restraint (only consisting of a subset of items included in the original but longer Cognitive Restraint subscale of the TFEQ-51), Uncontrolled Eating (consisting of a subset of items that were included in the original subscales Disinhibition and Hunger of the TFEQ-51) and Emotional Eating (including a subset of items from the Disinhibition scale of the TFEQ-51). The rationale behind the additional analysis of the TFEQ-R18 was an attempt to further specify the aspect of Disinhibition driving the effects observed with the TFEQ-51.

### Magnetic Resonance Imaging (MRI) Acquisition

T1-weighted images were acquired on a whole-body 3T TIM Trio scanner (Siemens, Erlangen, Germany) with a 12-channel head- array coil using a MPRAGE sequence [TI = 650 ms; TR = 1300 ms; snapshot FLASH, TRA = 10 ms; = 3.93 ms; = 10°; band-width = 130 Hz/pixel (i.e., 67 kHz total); image matrix = 256×240 = 256 mm×240 mm; slab thickness = 192 mm; 128 partitions; 95% slice resolution; sagittal orientation; spatial resolution = 1 mm×1 mm×1.5 mm; 2 acquisitions].

### Image Processing

SPM5 (Wellcome Trust Centre for Neuroimaging, UCL, London, UK; http://www.fil.ion.ucl.ac.uk/spm) was used for image pre-processing and statistical analysis. MR images were processed using the DARTEL approach with standard parameters for VBM running under MatLab 7.7 (Mathworks, Natick, MA, USA). All analyses were performed on bias-corrected, segmented, registered (rigid-body transformation), interpolated isotropic (1.5 mm×1.5 mm×1.5 mm), and smoothed (FWHM 8 mm) images. All images were warped based on the transformation of the group-specific DARTEL template to the GM prior image provided by SPM5 to meet the standard stereotactical space of the Montreal Neurological Institute (MNI). GM segments were modulated by the Jacobian determinants of the deformations introduced by normalization to account for local compression and expansion during transformation.

### Statistics on Demographic, Questionnaire and Imaging Data

#### Demographic & questionnaire data

Statistics on demographic and questionnaire data were performed using SPSS 19 (IBM). Because the assumption of normality was violated for most questionnaire data we used non-parametric tests (Kruskal-Wallis Test for independent samples and Mann-Whitney U-tests for *post hoc* pair-wise comparisons) on residuals after adjusting for age and BMI (within groups of men and women) of all scales save the ‘disinhibition’ subscale. We performed a one-way ANOVA including covariates for age and BMI for the subscale ‘disinhibition’ since values were normally distributed (Kolmogorov-Smirnov-test with Lilliefors significance correction: p = .099). α-level was.05 and all tests were performed two-sided and were adjusted for ties.

#### Imaging

A general linear model was estimated on the whole brain level with the factors genotype at rs17782313 and gender; age in years, BMI in kg/m^2^, total gray and total white matter volume in ml were included as covariates. Because of previously identified gender-specific relationships between GMV and BMI [18], we centered BMI on the mean of factor gender and included the interaction between BMI and gender in the model.

Based on our strong *a priori* hypotheses derived from animal experiments about the localization of structural alterations and the low minor allele frequency we accepted for the interaction effect on a whole-brain basis a mixed threshold of p<.005 on voxel-level and p<.05 on cluster-level, corrected for non-stationarity of smoothness. The main effect of rs17782313 within women was significant using a threshold of p<.001 on voxel-level and p<.05 on cluster-level, corrected.

## Results

**Figure 1:**
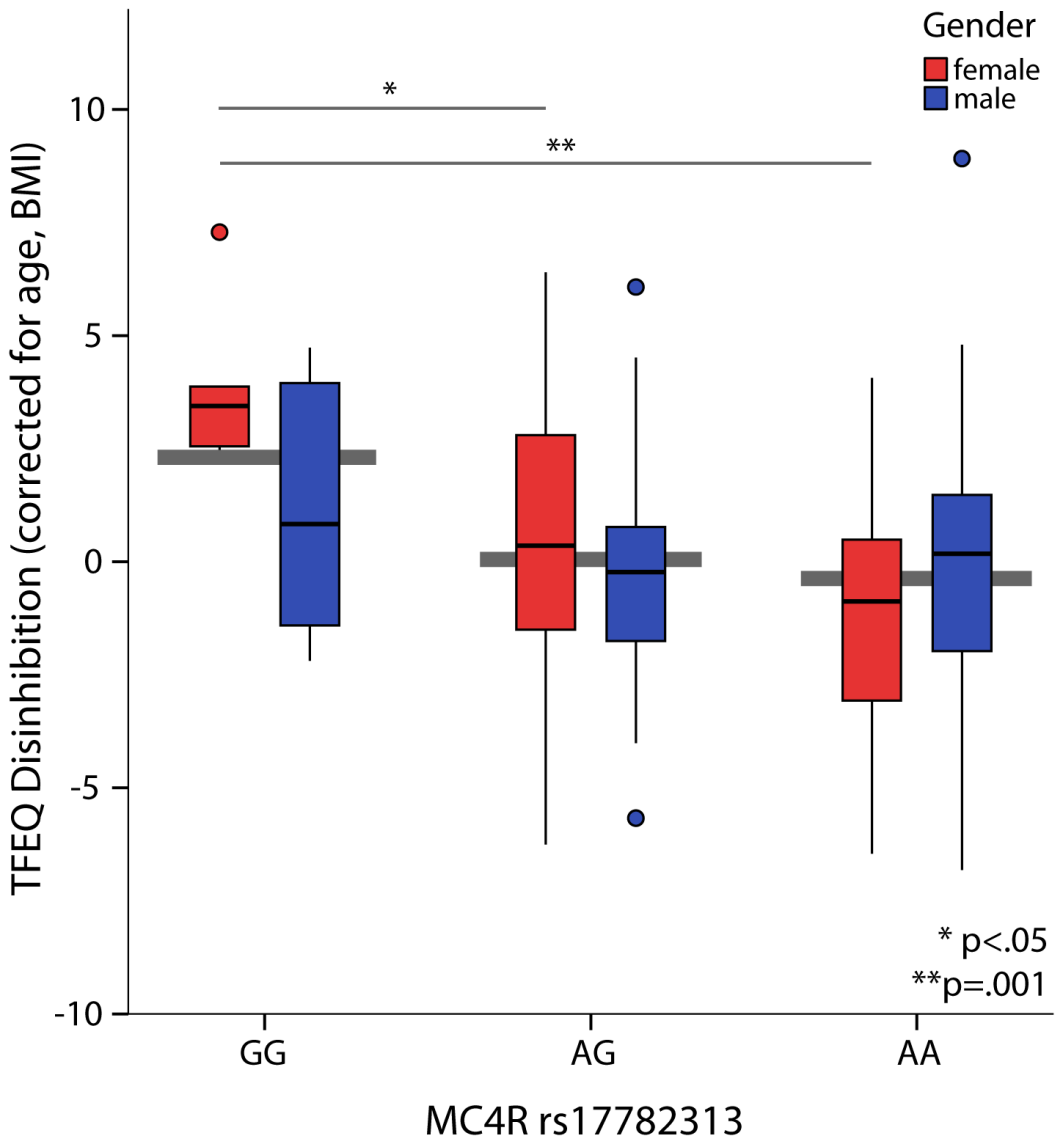
Sex-specific effect of rs17782313 near MC4R on ‘disinhibition’ scores (interaction F_2,177_ = 4.7, p = .01). Female homozygous carriers of the minor allele (CC) had significantly higher scores than both female heterozygous subjects (AC; difference in means 3.42, standard error 1.25, p = .024) and female subjects carrying no minor allele (AA; difference in means 4.93, standard error 1.22, p<.001). No significant difference was found for men (F_2,107_<1.5).

We found a significant association between rs17782313 and the ‘disinhibition’ score of the TFEQ-51 (main effect of rs17782313 F_2,177_ = 8.34, p<.001, partial η^2^ = .09, Table 1, Figure 1). Homozygous carriers of the minor allele (CC) scored higher than both heterozygous subjects (CA) and subjects carrying no minor allele (AA), suggesting a recessive mode of inheritance. Further, the interaction between genotype and gender was significant (F_2,177_ = 4.7, p = .01, partial η^2^ = .05). In addition, there was a main effect of gender (F_1,177_ = 19.37, p<.001, partial η^2^ = .1) and BMI that explained most of the variance (F_1,177_ = 48.06, p<.001, partial η^2^ = .21).

Next, we reanalyzed women and men separately to follow up on the observed interaction effect. Among female subjects only, we observed a significant effect of the genetic variant on ‘disinhibition’ scores (F_2,69_ = 8.98, p<.001, partial η^2^ = .21, Figure 1). Since there was no such effect for male subjects (F_2,107_<1.1, partial η^2^ = .02), the effect of rs17782313 on the whole sample is primarily driven by women. BMI explained most of the variance in both groups (Women: F_1,68_ = 27.12, p<.001, partial η^2^ = .29; Men: F_1,107_ = 21.80, p<.001, partial η^2^ = .17). Using the TFEQ-18 scoring scheme to further narrow the factor driving the effect observed for ‘disinhibition’ (i.e. shortened and revised version of the TFEQ, see [17] and methods section for further information), we moreover found a significant effect of rs17782313 on the ‘emotional eating’ subscale, again only in women (Kruskal-Wallis Test for independent samples and Mann-Whitney U-tests for *post hoc* pair-wise comparisons, H(2) = 12.66, p = .002; CC>AC: U = 24.38, z = 2.66, p = .023; CC>AA: U = 31, z = 3.52, p = .001, corrected for age and BMI).

**Figure 2:**
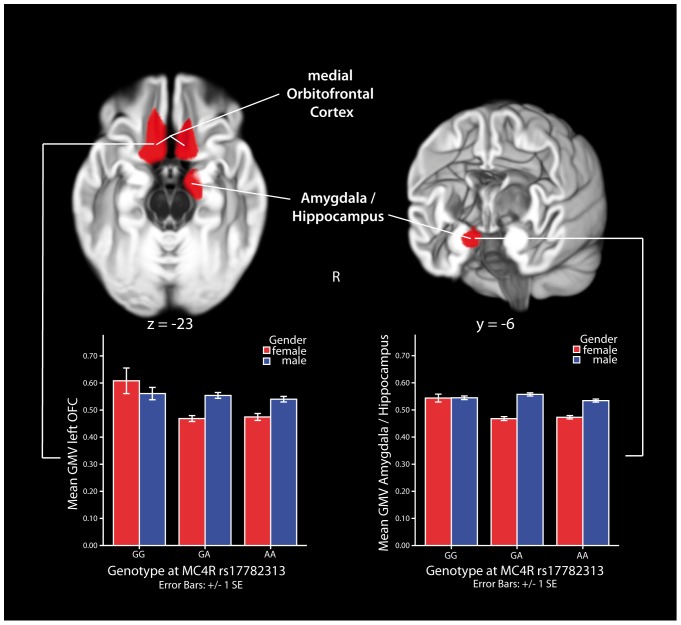
Sex-specific association between rs17782313 and gray matter volume (GMV). Sex-specific effects of genotype at rs17782313 on GMV in the right amygdala/hippocampus (z-value 3.97, x = 14, y = −6, z = −22) and bilateral medial orbitofrontal cortex (OFC; left OFC: z-value 3.95, x = −12, y = 24, z = −22; right OFC: z-value 3.76, x = 12, y = 20, z = −20), adjusted for participant’s BMI, age, global gray and white matter volume (interaction between gender and genotype, voxel-wise threshold p<.005, cluster threshold p<.05, corrected for non-isotropic smoothness, indicated by red color). Female homozygous carriers of the minor allele (CC) had higher values than heterozygous subjects (AC) or non-carriers (AA, voxel-wise threshold p<.001, cluster threshold p<.05, corrected for non-isotropic smoothness). No significant difference was found for men.

Next, in an extended sample of 221 subjects (fully including the previous sample, for details see Tables 1 & 2) we used voxel-based morphometry (VBM) of the whole brain to analyze the association of rs17782313 and gender with the brain’s gray matter volume (GMV). We found a significant interaction between genotype and gender on brain structure: In agreement with the lack of effects of rs17782313 on eating behavior in males there was also no association between rs17782313 and brain structure in males. In females, however, we found significant differences in a cluster comprising the right amygdala, a region known to influence, amongst others, ingestive behavior, and the anterior hippocampus, a structure crucially involved in memory formation and learning (Figure 2 left column). Further, we found bilateral differences in medial orbitofrontal cortex, a multimodal brain structure encoding the subjective value of reinforcers [19] (OFC, see Figure 2 right column) and left and right prefrontal cortex, a higher order regulation area. Female homozygous carriers of the minor C allele had higher GMV in these regions than females being heterozygous or carrying no minor allele (for a detailed description of all clusters see Table 3). Variations in GMV have been widely shown to correlate with functional variations of the same areas [20], [21].

**Table 3:** Detailed description of regions with a significant interaction between gender and rs17782313 near *MC4R*.

			MNI coordinates of maximum (mm)				
region	Brodmannarea	hemisphere	x	y	z	z-value	cluster extent (voxels)
amygdala (superficial nuclei group), entorhinalcortex, hippocampus	28, 35	right	14	−6	−23	3.96	999
orbitofrontal cortex, subcallosal cortex, posteriorgyrus rectus	11, 25	left	−10	22	−22	3.95	2168
subcallosal gyrus		left	−11	10	−17	3.89	
orbitofrontal cortex, subcallosal cortex, posteriorgyrus rectus	11	right	13	19	−20	3.76	827
prefrontal cortex, superior frontal sulcus	9	left	−27	21	36	3.69	1245
prefrontal gyrus, superior frontal gyrus, lateral part	46, 10	right	23	46	14	4.44	1268

**Figure 3:**
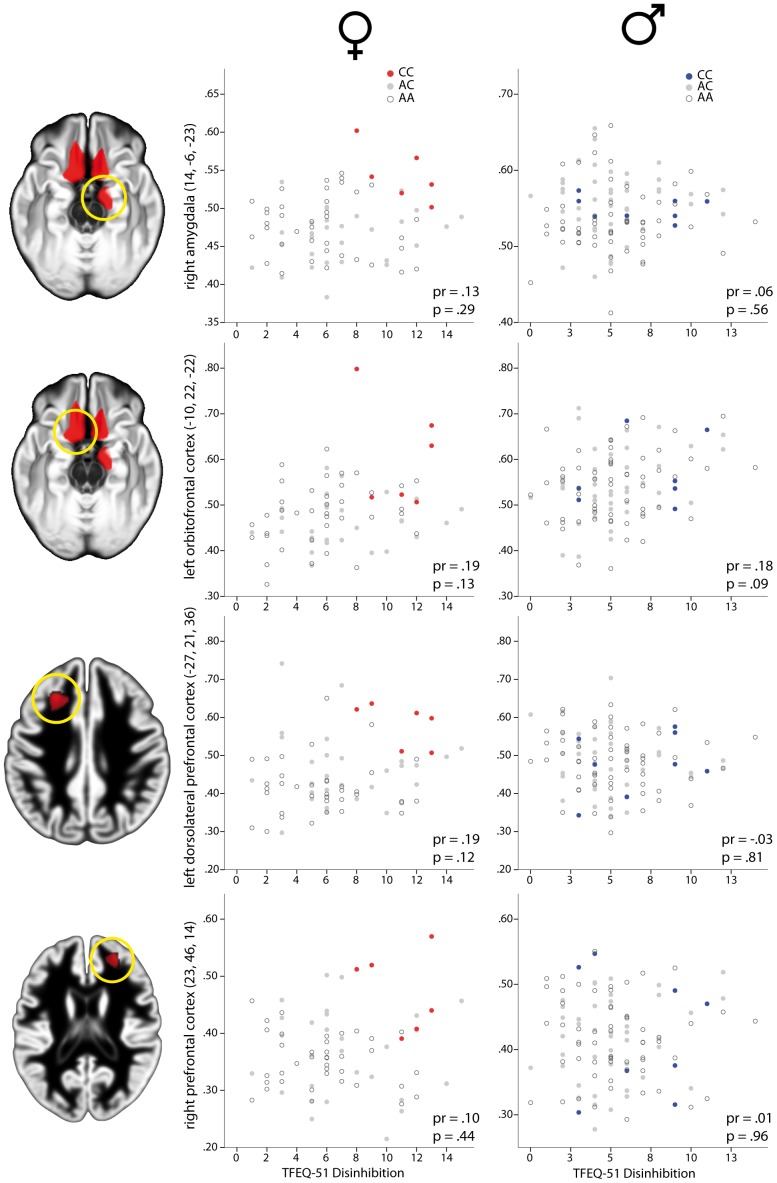
Distribution of gray matter volume and ‘disinhibition’ scores for brain areas showing a significant interaction between gender and genotype. Gray matter volume (GMV) in right amygdala (upper row), left orbitofrontal cortex (second row), left dorsolateral prefrontal cortex (third row) and right prefrontal cortex (bottom row) plotted against values on the ‘disinhibition’ subscale of the Three Factor Eating Questionnaire (TFEQ 51) for women (middle column) and men (right column) separately. Left column indicates the brain region where gray matter volume was extracted. Filled dark circles indicate homozygous carriers of the risk allele (CC) at rs17782313, filled gray circles indicate heterozygous subjects (AC) and open circles indicate homozygous carriers of the wildtype allele (AA). pr = partial correlation coefficient controlling for BMI, p = p-value of partial correlation analysis.

There was no direct and robust linear association between GMV and behavioral data (Figure 3).

## Discussion

Here, we report associations between common genetic variation near *MC4R* (rs17782313) and measures of eating behavior as well as human brain structure. For both, eating behavior as well as brain structure, we found differential associations with rs17782313 in women and men independent of BMI and age.

The reported effect of rs17782313 on eating behavior is in line with previous findings. Despite relying on different subjective measures of eating behavior, several studies have found reliable associations between variants near *MC4R* and excessive appetite [10].

rs17782313-associated alterations of brain structure in amygdala and OFC may commonly reflect changes in the development of conditioned feeding potentiation and state dependent reward response to ingested food [22] due to the prominent roles of these brain regions in reinforcement [23]. From animal experiments it is known that injection of MC4R selective antagonists into the central amygdala stimulates food intake [24]. In addition, lesions of the amygdala in rats alter macronutrient selection, while specific stimulation of MC4Rs in amygdala changes dietary preferences [22]. Furthermore, serotonin-induced hypophagia has been shown to require MC4-receptors [25] in amygdala and hypothalamus.

Interestingly, a recent fMRI study in normal-weight individuals found a differential relation between left and right amygdala function and cognitive restraint of the TFEQ on the one hand and self-directedness on the other [26]. Self-directedness refers to“the ability of an individual to control, regulate, and adapt behavior to fit the situation in accord with individually chosen goals and values”. While left amygdala activation in response to appetizing food pictures increased with increasing values of cognitive restraint, right amygdala activation increased with low levels of self-directedness. This suggests a high response of left amygdala in subjects who self-reported to regulate food intake with respect to individually chosen goals and values on the one hand and a high response of right amygdala of individuals lacking this ability to regulate and adapt their behavior, i.e. subjects prone to respond more reflexively to (external) triggers of eating.

Furthermore, functional magnetic resonance imaging (fMRI) experiments in women demonstrate that right amygdala and OFC demonstrate a heightened hemodynamic response to food under emotional stress [27]. Hence, these regions seem to be sensitive to stress and mediate an altered response to food in stressful situations. Therefore, the observed MC4R-related structural brain alterations may represent an altered layout of this pathway, ultimately leading to a functional change of this neurobiological mechanism. In the same vein, stress-induced anhedonia has recently been shown to depend on MC4R-mediated synaptic adaptations in the ventral striatum, the main reward relay of the brain [28].

Changes in the PFC are less straightforward to explain as this structure is most often reported to exert inhibition, also in the context of food intake. One possible interpretation of our results may be that rs17782313 primarily affects emotional eating, i.e. increased food intake when subjects are in negative mood, instead of modulating general disinhibition. Therefore, alterations in prefrontal structures might indicate compensatory mechanisms, which would go together with the notion that the genetic effect on emotional eating behavior in our sample is by far stronger than that on BMI. However, since we do not have functional brain imaging data of our participants, the direction of structural alterations is not conclusive on its own and further research is warranted.

Congruently with the gender specificity of our findings, two studies reported an association of the minor C allele of rs17782313 with higher BMI restricted to women [8], [16]. Liu and colleagues demonstrated in a sample of 1,649 children (mean aged 16.2 years) a robust interaction of the gene variant with sex [16]. However, in the study of Renström and colleagues (3,885 non-diabetic adults mean aged 52.6 years), only a nominally significant interaction with sex was observed, with the P-value not being robust to multiple statistical comparisons [8]. Further, Kvaloy and colleagues demonstrated a sex-specific temporal dynamic in the association between BMI and rs17782313 [15]. While the variant was inversely related to birth weight in boys (minor C allele associated with lower birth weight), this significant effect vanished at early adolescence and adulthood. In female newborns, the variant had no significant effect on birth weight. However, at early adolescence and adulthood the effect direction described for adults in other cohorts (minor C allele associated with higher BMI) emerged for females only, with the highest effect size at early adolescence. An attenuated trend in the same direction was observable for boys at early adolescence only. These data support the interpretation of the current findings, namely that the effect of rs17782313 might be stronger in women compared to men and that the effect might be mediated by different mechanisms in both genders. Unfortunately, our sample does not cover an age-range large enough to investigate possible age-related dynamics of rs17782313’s association with markers of obesity, eating behavior and brain structure.

Taken together, these data may point to an attenuated effect of rs17782313 in men compared to women with considerable age-related dynamics.

Our data seem to suggest a recessive mode of inheritance for rs17782313’s effect on eating behavior and brain structure in women. A recent meta-analyses on the association between common polymorphisms near the MC4R gene and obesity risk confirmed an association between rs17782313 and obesity under the assumption of each, additive, dominant, recessive and allelic models, with the highest odds ratio (OR) observed for a recessive model: OR of additive model 1.18, dominant model 1.26, recessive model 1.41 and allelic model 1.24 [29], and thereby providing supporting evidence for the plausibility of assuming a recessive mode of inheritance for this SNP in the current study.

### Limitations

The presented results should be regarded as an initial attempt to elucidate the central underpinnings of rs17782313’s effect on eating behavior. We are well aware of the limitations of the present study. Since the effect size for rs17782313 on BMI is known to be rather small (typically in the range of 0.01–0.02, see Loos et al. 2008), owing to a lack of power we did not expect to see a significant association between this SNP and BMI in our sample. We included data on BMI in Table 1 and 2 giving the BMI range for each subgroup. From these data it is clear that there is no significant effect of the variant on BMI in our relatively small sample, however a slight effect in the previously described direction detected in cohorts much larger than ours can be seen (see Table 1 and 2). As we cannot rule out other causes for an elevated BMI, we performed our analyses corrected for BMI to extract the direct effects of rs17782313 on eating behavior and brain structure. Further, with this lack of power the risk to overlook associations is necessarily high.

In the future, the identified association should be replicated in an independent cohort with a comparable age and BMI range. In addition, other SNPs near MC4R should be investigated with respect to their association with eating behavior and brain structure or function in humans in a large enough sample.

To conclude, the effect of common genetic variation near *MC4R* on eating behavior in women may be mediated through central mechanisms involving amygdala/hippocampus, medial OFC and prefrontal cortex. Further studies are needed to provide mechanistic insight in more detail and to corroborate the present findings.
